# Integrated modeling framework reveals co-regulation of transcription factors, miRNAs and lncRNAs on cardiac developmental dynamics

**DOI:** 10.1186/s13287-023-03442-0

**Published:** 2023-09-13

**Authors:** Shumin Li, Bin Yan, Binbin Wu, Junhao Su, Jianliang Lu, Tak-Wah Lam, Kenneth R. Boheler, Ellen Ngar-Yun Poon, Ruibang Luo

**Affiliations:** 1 Department of Computer Science, The University of Hong Kong, Pokfulam, Hong Kong China; 2 State Key Laboratory of Pharmaceutical Biotechnology, The University of Hong Kong, Pokfulam, Hong Kong China; 3 School of Biomedical Sciences, The Chinese University of Hong Kong, Shatin, Hong Kong China; 4 Centre for Cardiovascular Genomics and Medicine, Lui Che Woo Institute of Innovative Medicine, The Chinese University of Hong Kong, Shatin, Hong Kong China; 5 The Division of Cardiology, Department of Medicine and The Whiting School of Engineering, Department of Biomedical Engineering, The Johns Hopkins University, Baltimore, MD 21205 USA; 6 Hong Kong Hub of Paediatric Excellence (HK HOPE), The Chinese University of Hong Kong, Kowloon Bay, Hong Kong China

**Keywords:** Data integration, Cardiac development and function, Transcription factors, miRNAs, lncRNAs, Gene regulatory network

## Abstract

**Aims:**

Dissecting complex interactions among transcription factors (TFs), microRNAs (miRNAs) and long noncoding RNAs (lncRNAs) are central for understanding heart development and function. Although computational approaches and platforms have been described to infer relationships among regulatory factors and genes, current approaches do not adequately account for how highly diverse, interacting regulators that include noncoding RNAs (ncRNAs) control cardiac gene expression dynamics over time.

**Methods:**

To overcome this limitation, we devised an integrated framework, cardiac gene regulatory modeling (CGRM) that integrates LogicTRN and regulatory component analysis bioinformatics modeling platforms to infer complex regulatory mechanisms. We then used CGRM to identify and compare the TF-ncRNA gene regulatory networks that govern early- and late-stage cardiomyocytes (CMs) generated by in vitro differentiation of human pluripotent stem cells (hPSC) and ventricular and atrial CMs isolated during in vivo human cardiac development.

**Results:**

Comparisons of in vitro versus in vivo derived CMs revealed conserved regulatory networks among TFs and ncRNAs in early cells that significantly diverged in late staged cells. We report that cardiac genes (“*heart targets*”) expressed in early-stage hPSC-CMs are primarily regulated by MESP1, miR-1, miR-23, lncRNAs NEAT1 and MALAT1, while GATA6, HAND2, miR-200c, NEAT1 and MALAT1 are critical for late hPSC-CMs. The inferred TF-miRNA-lncRNA networks regulating heart development and contraction were similar among early-stage CMs, among individual hPSC-CM datasets and between in vitro and in vivo samples. However, genes related to apoptosis, cell cycle and proliferation, and transmembrane transport showed a high degree of divergence between in vitro and in vivo derived late-stage CMs. Overall, late-, but not early-stage CMs diverged greatly in the expression of “heart target” transcripts and their regulatory mechanisms.

**Conclusions:**

In conclusion, we find that hPSC-CMs are regulated in a cell autonomous manner during early development that diverges significantly as a function of time when compared to in vivo derived CMs. These findings demonstrate the feasibility of using CGRM to reveal dynamic and complex transcriptional and posttranscriptional regulatory interactions that underlie cell directed versus environment-dependent CM development. These results with in vitro versus in vivo derived CMs thus establish this approach for detailed analyses of heart disease and for the analysis of cell regulatory systems in other biomedical fields.

**Supplementary Information:**

The online version contains supplementary material available at 10.1186/s13287-023-03442-0.

## Introduction

Cardiogenesis is a dynamic developmental process that leads to the formation of a functional, contracting heart. The main force generating cells in mammalian heart, cardiomyocytes (CMs), arise from progenitor cells, originating in the primary and secondary heart fields. These progenitor cells express cardiac-restricted transcription factors (TFs) that regulate downstream gene programs [1, 2]. Among these are MESP1, which acts as a master regulator of cardiac lineage commitment [3, 4]. The cardiac TFs GATA4, GATA6, NKX2-5, MEF2A/C, SRF, ISL1, TBX5, HAND1 and HAND2 subsequently play critical roles in regulation of early embryonic cardiogenesis [5, 6]. The TFs HEY2 or NR2F2 and TBX5 direct ventricular or atrial identity [7, 8]. Any abnormalities in cardiogenesis associated with the dysregulation of these TFs can lead to congenital heart defects and dysfunctional CMs, which can contribute to heart failure, a leading cause of morbidity and mortality worldwide.

In addition to TFs, microRNAs (miRNAs) and long noncoding RNAs (lncRNAs) have important, but less well-characterized roles, particularly in human heart development. These noncoding RNAs (ncRNAs) modulate transcriptional and posttranscriptional activities necessary for cardiac commitment, differentiation and maturation [9–12] Developmentally, miRNAs such as miR-1, -133a, -199a, -208, let-7, -499 and -590 promote CM development and heart function, while miR-133a and -200c inhibit or repress CM development [13–16]. Some miRNAs facilitate reprogramming into CMs while others contribute to heart disease [13, 14, 16–18]. Similarly, lncRNAs like H19, CARMEN, MALAT1, NEG3, NEAT1, TUG1, GAS5 and MIAT have been implicated in the regulation of heart development, cardiac function and disease, but their roles and regulatory functions are still incompletely understood [9, 19, 20]. Ultimately, the interplay of TFs with ncRNAs control gene expression, the molecular foundation upon which cardiogenesis, CM development and heart maturation are regulated.

Human embryonic stem cells (hESC) or induced pluripotent stem cell (hiPSC)-derived CMs have served as an in vitro surrogate model of human development and maturation; however, recent publications suggest that in vitro derived hPSC-CMs undergo a developmental block which prevents them from recapitulating adult cardiac physiology. While this developmental block has been postulated to be regulated through transcriptional means [21, 22], the contribution of ncRNAs to CM development and a potential developmental block in vivo and in vitro are still poorly understood. A more complete understanding of the complex interactions among TFs and modulators of transcriptional and posttranscriptional control of gene expression as a function of time is essential to unraveling developmental processes of heart and for identifying regulators that may prevent the faithful recapitulation of development in vitro.

Computational methods and algorithms have been designed to infer gene regulatory networks (GRNs) through the integrated analysis of different types of biological omics data, such as ordinary differential equations, graphical Gaussians, Bayesian networks and matrix decomposition [23–25]. Web-based platforms and software with some of these functions have been developed, such as FFLTool [26], miRNACancerMAP [27] and PTHGRN [28], which are capable of identifying regulatory interactions among TFs, miRNAs or lncRNAs. These methods do not fully account for the dynamic and temporal nature of gene regulation. As an alternative, dynamic modeling and network approaches have been developed for the computational analysis of time-course omics data to dissect biomedical and biomedicine complex systems [29, 30], for example, TIMEOR (Trajectory Inference and Mechanism Exploration with Omics data in R) that builds transcriptional target networks by coupling predicted and observed TF-binding data [31]. These computational approaches, however, do not significantly improve upon existing predictions of GRNs over time. Consequently, it has proven extremely difficult to capture the dynamics of gene regulation by TFs and ncRNAs during developmental processes.

In this study, we developed an integrated web-based framework, cardiac gene regulatory modeling (CGRM), whose aim is to identify cross-interactions among TFs, miRNAs and lncRNAs to better understand how these factors control developmental progression of heart cells. CGRM incorporates two bioinformatics modeling based on LogicTRN [32] and regulatory component analysis (RCA) [33]. As a proof-of-principle, we used this modeling system first to analyze different stages of CMs differentiated in vitro from hPSC-CMs. We then extended these analyses to explore the regulatory landscapes of fetal embryo-derived atrial and ventricular CMs. The results from this study demonstrate that CGRM can reveal multi-level regulatory structures applicable to CM developmental dynamics that differ between in vivo and in vitro models. Specifically, we have identified regulatory networks present in hPSC-CMs that are conserved in early stages, but which diverge from networks identified in late stages of development. These findings have implications for basic and applied research applications requiring hPSC-CMs and for any potential future therapeutic application of hPSC-CMs, which may require more mature CMs.

## Methods

### Time series genome-wide expression data of mRNAs

The processed or raw count data of time series genome-wide microarrays and RNA-seq expression data of human CMs were extracted from GEO/NCBI (Table 1). Based on the accessible datasets for in vitro differentiated CMs, we divided the data into groups corresponding to differentiation and to maturation stages. From time points of in vitro hPSC-CMs, we defined the early CMs from differentiation days (D) 0–14 or 15. From D0-D7, hPSCs differentiate to mesoderm and early cardiac progenitors that give rise to very immature hPSC-CMs by D6–D8. Between D7 and D15, the cells are committed to the cardiac lineage but retain proliferative potential and are commonly considered to be immature and embryonic-like. We defined late CMs after D15. During this phase, the cells stop dividing and undergo some degree of maturation.

**Table 1:** Datasets of time series genome-wide expression of mRNAs during in vitro CM differentiation and in vivo CM development

GEO accession number	Proposed stages	Materials
GSE76523	Early (0, 1, 2, 3, 4, 5, 6, 8, 10, 15 days)	H7 human embryonic stem cell-CM differentiation
GSE239918	Late (15, 30, 45, 60 days)	
GSE81585	Early (0, 1, 2, 3, 4, 5, 6, 7, 8, 9, 14 days) and Late (14, 30, 90 days)	Human-induced pluripotent stem cell-CM differentiation
GSE35671	Early (0,3,7,10,14 days) and Late (14, 28, 45, 60, 90, 120 days)	
GSE106118	Early (5, 6, 7, 9, 10 weeks) and Late (13, 15, 17, 20, 22, 23, 24, 25 weeks)	Human fetal embryos-atrium and ventricle development

The in vivo embryo-CM data (accession number GSE106118) utilized in this study were extracted from 13 time-point single cell RNA-sequencing experiments from 5 to 25 weeks (W) of gestation [34]. Among the cardiac cells, atrial and ventricular samples are two main types, which have been designated as early (E) and late (L) stages of atrial (A) and ventricular (V) CMs. Two time periods, 5W–10W and 13W–25W, were used to represent the early and late stages, respectively. The mRNA expression data from the four subgroups, atrial (CM-AE, CM-AL) and ventricular (CM-VE and CM-VL) CMs, were separately analyzed further.

### Data processing of mRNA expression in human CMs

Differentially expressed genes/mRNAs (DEGs) were analyzed using the downloaded raw counts, TPM or FPKM for RNA-seq and the normalized microarray datasets described in Table 1. We performed a statistical analysis to identify DEGs between different time points at early and late stages of each dataset. For data with raw counts, we used Edge-R, and other data formats were analyzed using the limma R package. Fold changes of mRNA expression at different time points were calculated. DEGs were set at a cutoff of fold change at least 2.0 and *p* value below 0.05.

### Identification of TF logics and target genes through LogicTRN modeling

The LogicTRN model quantitatively infers logic relations between TFs by combining *cis*-regulatory logics and transcriptional kinetics in a single-model framework. This model integrates a time series gene expression profile and TF-DNA binding information to decipher TF regulatory logics in gene transcription [32]. In brief, the logicTRN model treats the dynamic gene expression profile as a process controlled by TF-binding occupancies and kinetic parameters (Eq. 1). To dissect the relationships of activation/inhibition among TF-TF pairs, LogicTRN integrates TF Boolean logic into the framework and infers the most likely TF cooperation on the target genes. The confident TF logics were predicted by fitting a regression on the groups of model equations of all possible TF regulatory logics. In this study, TF logics with confidence ≥ 0.9 were selected.

1 $$I_{{\text{s}}} \left( t \right) = I_{\max } \mathop \sum \limits_{n = 1}^{\infty } \left( {\left( { - 1} \right)^{n + 1} \left( {\frac{{k_{{\text{b}}} }}{{I_{\max } }}} \right)^{n} Y^{n} \left( {t - T_{m} } \right)/n!} \right)$$

where *I*_max_ denotes the initial transcription rate, *T*_*m*_ means the time delay of the TF regulation effect on the transcription, and *T*_*m*_ was set as 1 in this study. *k*_b_ is the TF activation strength, and Y indicates the TF-binding strength to the target gene, which is calculated from TF-binding signals. Note that in the algorithm, *k*_b_ and Y can be calculated and substituted based on different TF logics and the binding site identities or enrichment scores ChIP-seq or TF-gene binding data.

The confidence of TF logics can be inferred by following two steps, 1) estimating the matching probability between TF logics and TF combinations, and 2) inferring the confidence score of a TF logic based on the matching probabilities with all the TF combinations. Therefore, we can obtain the most likely TF logics based on the confidence score.

To run LogicTRN on the framework, two inputs, time series gene expression and TF-DNA binding data, are required. Default settings are provided, but some parameters can be changed, such as the TF regulation effect time delay and logic confidence threshold. Three types of regulatory logics, AND, OR and NOT are used to represent the interaction of TFs, respectively, in concurrent, independent and inhibitive operations. In the outputs, we use &, | and > to represent AND, OR and NOT logic, respectively. For example, “TF1 & TF2” refers to common action by the two TFs, “TF1|TF2” to either TF1 or TF2 action, and “TF1 > TF2” to TF1 prior to TF2.

### Identification of regulator–target interactions through RCA modeling

To infer interactions between regulators and targets, the RCA model performs regulator module identification based on a matrix decomposition approach through matrix factorization [33]. This method can profile the regulatory interactions of each regulator with all targeted mRNAs or ncRNAs, without the value ranging from 0 (i.e., non-interaction) to above. In brief, the overall process of RCA minimizes the loss function (shown in Eq. 2) for obtaining the regulator-gene activity strength matrix.

2 $$L = \left| {\left| {{\mathbf{X}} - {\mathbf{YZ}}} \right|} \right|$$

where **X** is the gene expression matrix and **Z** is the regulator–target activity profile, which is built from input regulator–target data and **X**, based on the median (or other parameters) expression values of the regulator targets. **Y** is the output indicating the predicted regulator-gene interactive strength. Through 1000 permutation tests, we estimated how likely true the regulator–target interactions were by comparing the random results with **Y**. Note that input expression data (i.e., the **X** matrix) are recommended to separate up-regulated and down-regulated groups when running RCA. In this study, we performed RCA to identify interactions between miRNAs or lncRNAs and the targeted mRNAs. Users can also adjust the parameters. We also provide multiple methods to construct the regulator–target activity profile with the median value as the default option.

### Evaluation of the interaction and association among gene sets

CGRM is equipped with software and statistical tools to quantify the interactive similarity among gene sets. By calculating the multiple association indexes, the framework evaluates whether the resulting interactions are significantly enriched between the target genes of different regulators, heart disease and functional gene sets. These methods include a connection specificity index [35], a Jaccard index, a Simpson index and a hypergeometric *p* value of over-representation test. The *p* values of the enrichments are used to evaluate whether target gene sets of TFs, miRNAs, lncRNAs or combinations of these regulators are significantly associated with functional or disease gene sets. The input data can be the target genes of different regulators (TFs, miRNAs and lncRNAs) or various gene sets extracted from our “Resource” (see the website http://www.bio8.cs.hku.hk/CGRM/) or from user-defined.

## Results

### Cardiac gene regulatory modeling (CGRM) framework

In this study, we postulated that complex gene regulatory mechanisms are driven by interplays of TFs, miRNAs and lncRNAs that involve both transcriptional and posttranscriptional activities. To uncover these mechanisms, we have incorporated two computational models LogicTRN and RCA into CGRM. The time series expression data of mRNAs are used as input, coupled with input regulatory factor-gene/mRNA interaction data extracted from published datasets, and experimental or computational analyses (see Additional file 1). LogicTRN identifies regulatory logics formed by the fourteen cardiac TFs and the target genes that represent TF-TF cooperative regulation on target genes and the underlying transcriptional regulatory networks across time. RCA identifies the most likely interaction modules between miRNA/lncRNA and target gene/mRNA through matrix decomposition and factorization. The outputs obtained from the two models provide a basis to define regulatory interrelationships over time among TFs, miRNAs, lncRNAs and their targets from which temporal GRNs in different time periods can be constructed.

**Fig. 1:**
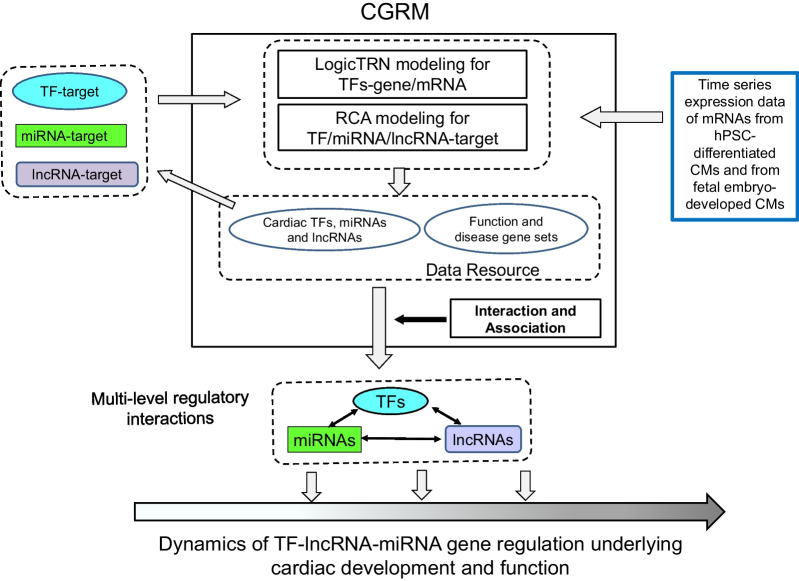
Overview of the web-based framework CGRM. CGRM establishes the cooperative regulation of TFs, lncRNAs and miRNAs on cardiac developmental dynamics. The main components of CGRM include computational modeling, interaction and association, and data resources

CGRM also incorporates software and statistical tools into “Interactions and Associations” that can calculate the enrichment of interactions among the target gene sets. The detailed procedure for using CGRM, selecting input data and the modeling parameters, can be found on the tutorial page of the website (http://www.bio8.cs.hku.hk/CGRM/). Figure 1 displays a schematic and an overview of CGRM in graphic form.

### Co-regulation of TFs with ncRNAs on hESC/hiPSC-CM differentiation

To conduct proof-of-principle studies with this framework, we applied CGRM to analyze input datasets both from in vitro differentiated CMs derived from hESC/hiPSCs and in vivo derived CMs from atrium and ventricle. By studying both cell origins, the goal was to identify regulatory associations and biological functions that were conserved among TFs and ncRNAs during dynamic processes of in vitro differentiation and in vivo development.

#### TF-TF

**Fig. 2:**
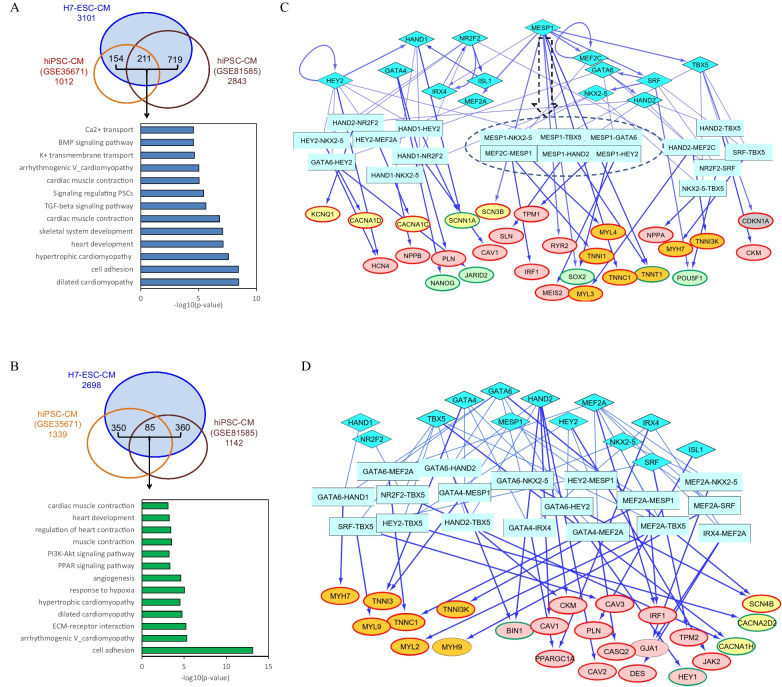
Transcriptional gene regulation of hPSC-CM differentiation and maturation. **A** and **B** Comparison of the differentially expressed gene targets of cardiac TFs which were predicted by using three datasets of hESC/hiPSC-CMs and the significantly associated functions at the early stage (**A**) and late stage (**B**). **C** and **D** Cardiac TF-gene regulatory networks at the early (**C**) and late stages (**D**). Oval nodes represent the target genes of TF logics and are involved in heart development and function (in light red), contraction (in orange), ion transport and handling (in yellow) and stem cell pluripotency regulators (in light blue). Oval nodes with red and green borders indicate up- and down-regulated genes, respectively. The arrow edges indicate regulatory relationships of TF logics and target genes, and the thick edges indicate that the regulatory relations between TFs and target genes are conserved in both hESC-CMs and hiPSC-CMs

To investigate the transcriptional control of cardiac developmental dynamics, we first focused on hESC-CMs generated with the H7 cell line across ten early-stage and four late-stage time points. Data from two other hiPSC lines were analyzed to exclude interline variability of regulatory networks among lines. The fourteen cardiac TFs used in LogicTRN are believed to play critical roles in modulating cardiac differentiation and specification, and heart development (Addition file 1). Dynamic changes in mRNA expression of these TFs during differentiation of hESC/hiPSC-CMs were observed (Additional file 3: Fig. S1). Particularly in early stages, significantly increased levels of MESP1, GATA4, GATA6, HAND1, NKX2-5, TBX5, etc., were detected in all lines examined. Additional file 2: Tables S1–S3 provide the predicted TF logics and the DEG targets using the three datasets of in vitro differentiated CMs. We then identified “conserved targets,” which we defined as target genes that are regulated by at least one common TF and are detected both in the hESC dataset and at least one of two hiPSC datasets. By intersection of TF-target genes generated from the three datasets, we obtained a total of 1084 and 795 conserved targets in corresponding early and late CMs. Subsequent functional annotations showed that the targets were significantly associated with critical cardiac biological processes and pathways. In early CMs, these functions included heart development, K^+^ and Ca^2+^ signaling, muscle contraction and some cardiac diseases like dilated and hypertrophic cardiomyopathy. In late CMs, additional functions involving PPAR, ECM and PI3K-AKT signaling, and the response to hypoxia were prevalent (Fig. 2A, B). As these latter results are consistent with a more mature phenotype, these data support our use of “early” versus “late” CMs to define temporal regulatory logics.

Among the top 50 predicted logics of the cardiac TFs, MESP1 logics had the largest number of target genes during the early stages of H7-CM differentiation (Additional file 2: Table S4). We proceeded to examine target genes involved in heart-related biological processes, pathways and disease, defined as “*heart targets*.” MESP1 was found to contribute to the largest number of either total or conserved *heart targets* in early CMs (Table 2). However, its contribution to the regulation of *heart targets* in late CMs is reduced.

**Table 2:** Heart target genes of cardiac TFs during hESC/hiPSC-CM differentiation

TF	Number of TF-target genes at early stage				Number of TF-target genes at late stage			
	H7-ESC-CM	hiPSC-CM (GSE81585)	hiPSC-CM (GSE35671)	Conserved target	H7-ESC-CM	hiPSC-CM (GSE81585)	hiPSC-CM (GSE35671)	Conserved target
GATA4	51	52	33	28	54	38	14	31
GATA6	120	120	114	71	198	99	30	90
HAND1	91	75	40	46	110	54	9	53
HAND2	101	77	31	59	138	62	51	58
HEY2	130	120	78	85	111	55	26	51
IRX4	20	9	5	11	35	4	6	8
ISL1	25	22	12	16	19	12	12	10
MEF2A	67	67	63	42	89	61	39	51
MEF2C	39	40	4	26	29	37	13	12
MESP1	188	176	100	103	162	82	45	86
NKX2-5	83	50	25	39	56	62	24	34
NR2F2	94	89	47	58	27	124	44	16
SRF	85	94	78	36	121	74	66	66
TBX5	95	61	43	54	111	84	44	64

The “conserved target” is the target genes that are regulated by at least one common TF, and are detected by H7-ESC dataset and at least one of two hiPSC datasets (GSE81585 and GSE35671)

Next, we constructed temporal cardiac transcriptional GRNs corresponding to the early and late stages of CMs (Fig. 2C, D). The early GRN exhibits a dominant role of MESP1 that was predicted to cooperate with several other cardiac TFs to drive the up-regulation of *heart targets* related to development (*IRF1*, *RYR2* and *SLN*), muscle contraction (*MYL4*, *TNNT2* and *TNNC1*) and ion transport and handling (*KCNQ1 CACNA1C* and *SCN3B*), and to down-regulate stem cell pluripotency regulators (*POU5F1* and *SOX2*). In late CMs, cardiac TFs HAND2, GATA6, SRF, NR2F2 and TBX5 are predicted to regulate a large set of downstream *heart targets* (Table 2). These TFs appear to co-activate target genes important for cardiac function (Fig. 2D). For example, the predicted up-regulation of *PPARGC1A* in late stages is validated by the previous report that in vitro dysregulation of PPARGC1A contributes to a developmental block that impedes CM maturation [36]. Our findings support the known role of MESP1 in early cardiac development and lineage specification and the roles of the other cardiac TFs to control later stages of cardiac development and function. The comparative data between hESC-CM and hiPSC-CM are also in accord with the identification of conserved cardiac transcriptional regulatory programs.

#### TF-miRNA

To identify interactions between TFs and miRNAs in regulation of CM differentiation processes, we utilized the RCA model to identify putative target genes of seven selected miRNAs (miR-1, -133a, -199a, -208, -23, -499a and -590) important for promoting cardiac development and function (Additional file 1). According to the predicted DEG targets of these miRNAs from the same three hESC/hiPSC-CMs (Additional file 2: Tables S1–S3), we identified cardiac TF-miRNA co-regulated targets and determined that ~ 30 to 40% of them are *heart targets*.

**Fig. 3:**
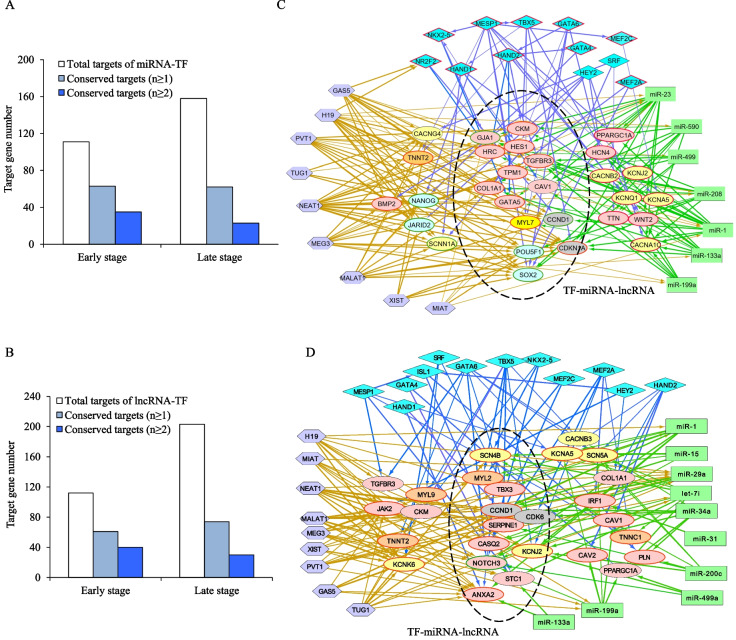
Target genes co-regulated by cardiac TFs, miRNAs and lncRNAs during hPSC-CM differentiation and maturation. **A** and **B** The percentage of the conserved targets of miRNA-TF or lncRNA-TF among all heart targets of hESC-CMs at the early (**A**) and late (**B**) stages. The “conserved targets” refer to those that are commonly regulated by at least one (*n* ≥ 1) or two (*n* ≥ 2) shared miRNAs or lncRNAs in both hESC-CMs and hiPSC-CMs. **C** and **D** The cardiac gene regulatory networks (GRNs) of TF-miRNA-lncRNA at the early (**C**) and late (**D**) stages. Oval nodes represent the target genes of TFs and miRNAs or lncRNAs, and are involved in heart development and function (in light red), contraction (in orange), ion transport and handling (in yellow), apoptosis (in gray) and stem cell pluripotency regulators (in light blue). Oval nodes with red and green borders indicate up- and down-regulated genes, respectively. The arrow edges indicate regulatory relationships of TFs and miRNAs or lncRNAs on target genes, and the thick edges indicate conserved regulatory relations detected in both hESC-CMs and hiPSC-CMs

Like TFs, we defined “conserved miRNA targets” to be genes regulated by at least one common miRNA both in hESC-CMs and in hiPSC-CMs. Of these, 56.7% or 31.5% of the *heart targets* are likely co-regulated by cardiac TFs and by at least one or two common miRNAs in early hESC-CMs (Fig. 3A), especially miR-1 and -23 that have more conserved targets (Table 3). We determined that miR-1, -23 and -133a or -590 likely interact frequently with cardiac TFs to regulate *heart targets* in early CMs. Among the analyzed cardiac TFs, MESP1, HEY2, GATA6 and HAND1 cooperate with the miRNAs at the highest frequency (Additional file 2: Table S5). Their interactions are significantly enriched in early differentiation (Additional file 3: Fig. S2).

**Table 3:** Heart target genes co-regulated by ncRNAs (miRNAs and lncRNAs) and cardiac TFs during hESC/hiPSC-CM differentiation

ncRNAs	Number of heart target genes (early stage)				ncRNAs	Number of heart target genes (late stage)			
	H7-CMs	hiPSC-CMs (GSE81585)	hiPSC-CMs (GSE35671)	Conserved targets		H7-CMs	hiPSC-CMs (GSE81585)	hiPSC-CMs (GSE35671)	Conserved targets
miR-1	57	56	59	27	let-7i	74	22	15	12
miR-133a	39	67	51	20	miR-1	55	29	28	14
miR-199a	41	57	30	17	miR-133a	51	34	24	10
miR-208	40	39	49	15	miR-15	95	33	33	14
miR-23	81	75	50	29	miR-199a	61	21	23	10
miR-499a	39	56	46	17	miR-200c	90	43	26	17
miR-590	60	57	34	17	miR-29a	46	26	29	15
					miR-31	42	35	23	11
					miR-34a	61	39	35	19
					miR-499a	76	39	17	12
GAS5	43	55	64	18	GAS5	67	57	17	9
H19	42	47	63	16	H19	47	63	8	12
MALAT1	60	107	38	24	MALAT1	83	32	38	26
MEG3	30	123	40	14	MEG3	94	19	8	9
MIAT	19	48	20	2	MIAT	69	43	18	5
NEAT1	82	94	85	40	NEAT1	101	39	63	40
PVT1	49	62	23	17	PVT1	52	14	11	6
TUG1	40	60	30	14	TUG1	69	68	10	8
XIST	27	50	17	8	XIST	51	26	3	2

The “conserved target” is the target genes that are regulated by at least one common miRNA or lncRNA, and are detected by H7-ESC dataset and at least one of two hiPSC datasets (GSE81585 and GSE35671)

In late-stage CMs, we focus on ten miRNAs (let-7i, miR-1, -133a, -15, -199a, -200c, -29a, -31, -34a and -499a) predicted to be important for cardiac development and maturation (Additional file 1). The subsets of *heart targets* co-regulated by the selected TFs and miRNAs differ among the three CM datasets (Table 3). The conserved *heart targets* of the ten miRNAs in H7-CMs are decreased to 39.2% or 14.6% for at least one or two common miRNAs (Fig. 3A) and also for every miRNA (Table 3). GATA6, HEY2, MESP1, HAND1 and HAND2 were predicted to be the main TFs that interact with let-7i, miR-15, -200c and -499a (Additional file 2: Table S5, Additional file 3: Fig. S2). By contrast, in hiPSC-CM datasets, the main TFs are SRF, GATA6, NR2F2, HAND2 and MEF2A that jointly work with miR-200c, -34a, -133a or -15, suggesting potential interline variability in the control of late CM development. The number of total and conserved targets of the TF-miRNA decrease from early to late stages, consistent with divergent TF-miRNA gene regulatory programs during the process of cardiac maturation.

#### TF-lncRNA

We then employed the RCA method to predict DEG targets of nine pre-defined lncRNAs known to be important for heart development and function (Additional file 1). The predicted target genes of these lncRNAs (Additional file 2: Tables S1–S3) were used to unravel co-regulation of cardiac TFs and the lncRNAs (Additional file 2: Table S6). Among these, lncRNAs, NEAT1, MALAT1, MEG3 or GAS5 were predicted to have the largest number of *heart targets* in common with cardiac TFs in the early-CMs (Table 3). The proportion of the conserved *heart targets* regulated by cardiac TF and at least one or two common lncRNAs was 55.4% or 35.7% (Fig. 3B), in particular NEAT1 and MALAT1 (Table 3). But in late stage, such proportion decrease to only 36.4% or 14.7% (Fig. 3B).

In the late-stage CMs, the number of *heart targets* of TF-lncRNA displays a divergent trend, with an increase in hESC-CMs and decrease in hiPSC-CMs (Table 3). The enrichment analysis shows significant overlap between the target genes of lncRNAs NEAT1, MALAT1, MEG3 and MIAT with cardiac TFs (Additional file 3: Fig. S3). Overall, a large divergent interplay between the heart targets of TF-lncRNAs was observed in the late-stage hESC-CMs and hiPSC-CMs (Additional file 3: Fig. S3).

#### TF-lncRNA-miRNA

By identifying the interplays among TFs, lncRNAs and miRNAs predicted to regulate *heart target*s, we have been able to construct early- and late-stage cardiac GRNs (Fig. 3C, D). From the early GRN, we observed a striking role of MESP1 in linking miRNAs and lncRNAs to promote CM differentiation (Fig. 3C). Overall, cooperation among the three types of factors is predicted to contribute to the up-regulation of *heart target*s, such as *CDKN1A*, *CKM*, *COL1A1*, *MYL7*, *HRC*, *TGFBR3*, *HES1* and down-regulation of pluripotent TFs *SOX2* and *POU5F1*. The cardiac TFs also work with miRNAs to regulate *CACNA1C*, *KCNA5*, *KCNQ1* and *KCNJ2*, *GJA1* and *HCN4*, and with the lncRNAs to regulate *TNNT2*, *BMP2*, *NANOG* and *JARID2*, which are important for cardiac development and function. In the late GRNs (Fig. 3D), we also identified the *heart targets* co-regulated by TF-miRNA-lncRNA, including those associated with ion transport, metabolism and contraction such as *MYL2*, *TBX3*, *CKM*, *KCNJ2*, *SCN4B*, *SERPINE1* and *CASQ2*. However, some targets (e.g., *CD36*, *CDKN1A*, *CKM*, *KCNJ2*, *KCNA5* and *TNNT2*) were up-regulated by different combinations of TFs and ncRNAs in early and late CMs. This analysis thus reveals previously unrecognized, complex regulatory mechanisms driven by interplays of TF-lncRNA-miRNA to control transcript levels that likely contribute to dynamics of CM differentiation and maturation.

### Application of CGRM to in vivo human embryonic cardiac development

**Fig. 4:**
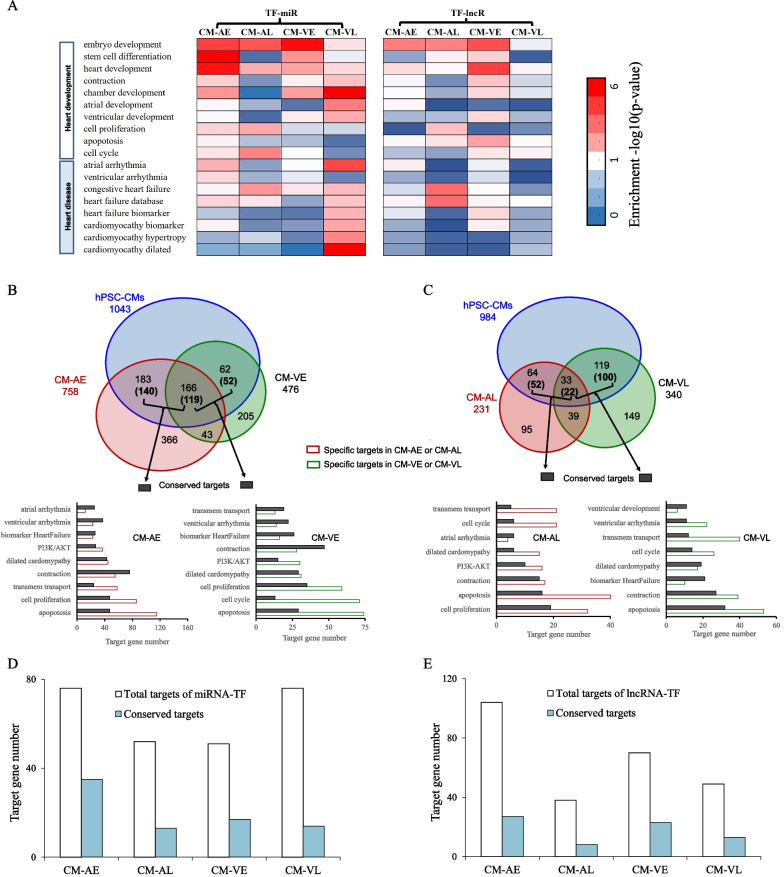
Gene co-regulation of TFs, miRNAs and lncRNAs on the early and late stages of in vivo CM development. **A** Enrichment analysis of the co-regulated genes of TF-miRNA and TF-lncRNA related to heart development, function and disease during in vivo fetal embryo-derived atrial and ventricular CM development. The p values represent significance of the enrichment analysis based on hypergeometric distribution. **B** and **C** Comparison of the differentially expressed heart genes regulated by the cardiac TFs, miRNAs or lncRNAs. The datasets of hPSC-CMs and fetal embryo-derived atrial and ventricular CMs at the early stages (**B**) and late stages (**C**) were used. The number in the Venn diagram shows the number of *heart targets* shared by the different datasets. The numbers of “conserved heart targets” shown in the parentheses indicate how many target genes are regulated by at least one common TF, miRNA or lncRNA between hPSC-CMs and embryo-derived CMs, i.e., CM-AE, CM-AL, CM-VE and CM-VL. The associated pathways, processes and heart diseases with the conserved and specific target gene programs are shown correspondingly. The black bars correspond to conserved targets, and the open bars are those that are specific to either CM-AE, CM-AL, CM-VE and CM-VL (i.e., not conserved relative to hPSC-CMs). **D** and **E** The percentage of the conserved targets of miRNA-TF (**D**) and lncRNA-TFs (**E**) in total heart genes of hESC-CMs at the early and late stages

We extended the application of CGRM to in vivo human embryonic cardiac development, ultimately to identify complex TF-miRNA-lncRNA target genes conserved in differentiated CMs. Our analysis of input mRNA expression data from in vivo CMs identified DEG targets in four subgroups, early (CM-AE and CM-VE) and late (CM-AL and CM-VL) stages that were strongly associated either with heart development or with heart disease (Additional file 2: Tables S7–S8). These regulatory associations were significant when analyzed using CGRM’s statistical tools (Fig. 4A). These results are consistent with an interplay of TFs and ncRNAs in the regulation of embryonic differentiation and heart formation during the early in vivo cardiac development.

A high proportion of *heart targets* were found to be regulated mainly by logics of GATA6, HEY2, NR2F2, HAND2 or TBX5 in early atrial and ventricular CMs (Additional file 2: Table S9). Cardiac TFs during late stages regulate far fewer *heart targets* in CM-AL and CM-VL relative to the early stages (Additional file 2: Tables S9 and S10). Comparisons of the ventricular and atrial samples show that HEY2, a TF known to control ventricular identity, was predicted to regulate large numbers of DEGs in CM-VE, while atrial-prevalent TFs such as NR2F2 regulate big number of DEGs in CM-AEs (Additional file 2: Table S10). Both ventricular- and atrial-prevalent TFs regulate many more DEGs during early stages when compared to late stages.

We used CGRM to identify those *heart targets* that are similarly regulated during in vitro CM differentiation and in vivo CM development. Our goal was to identify those regulatory programs that are conserved between the two different CM sources, which may be reflective cell autonomous mediated regulatory mechanisms. Figure 4B, and C show the *heart targets* that are predicted to be regulated jointly by cardiac TFs, miRNAs and lncRNAs. Approximately 80% of the intersected targets are predicted to be regulated by at least one common factor among TFs and ncRNAs in the four CM subgroups. During early stages, 259 and 171 *heart targets* in atrial and ventricular CMs, respectively, were also present in the hPSC-CM datasets, with 119 targets shared by both (Fig. 4B). A majority of the conserved targets involves contraction, heart development and diseases. By comparison, CM-AL and CM-VL shared far fewer conserved target genes (74 and 122, respectively) with in vitro derived hPSC-CMs in late stages (Fig. 4C). Unlike the early stages, most genes involved in contraction and cardiac diseases are specific to in vivo samples and are not conserved relative to hPSC-CMs during late stages (Fig. 4B, C). The most divergent targets correspond to genes related to apoptosis, cell cycle & proliferation and transmembrane transport. This divergence is notable among both early- and late-stage CMs, indicating that these processes are poorly conserved between CMs in vitro and in vivo. We also compared the conservation among different types of regulators. Among TF-miRNA targets, a high degree of conservation was observed among in vitro and in vivo derived CMs, especially those from early stages [CM-AE (46.1%) and CM-VE (33.3%)] compared to late stages [CM-AL (25.0%), vs. CM-VL (18.4%)] (Fig. 4D). In contrast, the number of the conserved targets of TF-lncRNA is lower than that of TF-miRNA (Fig. 4E), indicating more divergent target genes of TF-lncRNA.

**Fig. 5:**
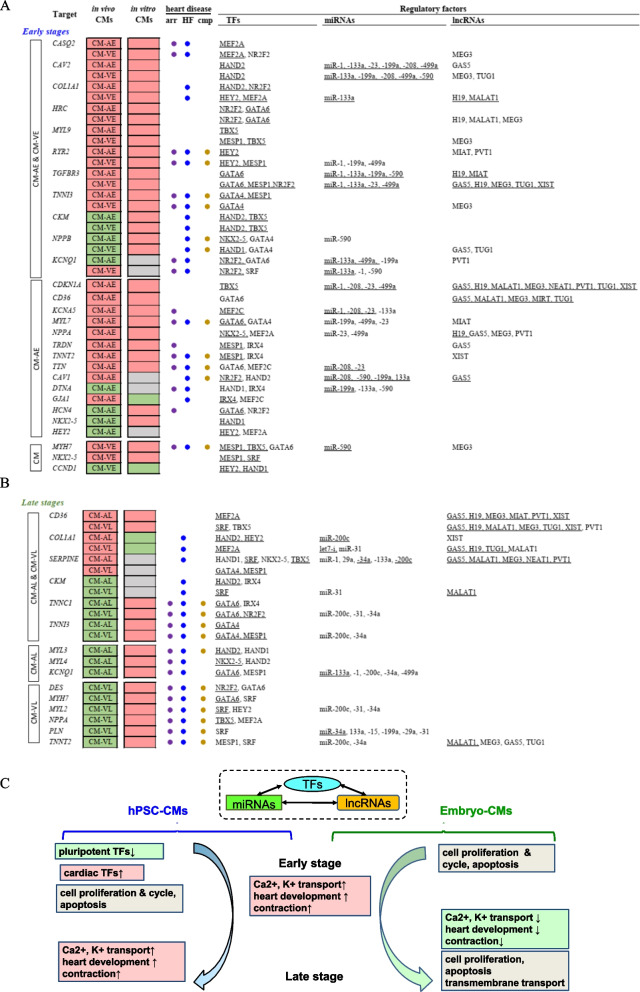
Cardiac gene regulatory programs of TFs, miRNAs and lncRNAs in fetal embryo-developed atrial and ventricular CMs. **A** and **B** comparison of heart target genes generated from in vivo atrial and ventricular CMs with in vitro hPSC-CM data during the early (**A**) and late stages (**B**). The listed differentially expressed gene targets show the conserved regulatory programs between both in vivo and in vitro and the association with heart development and disease. Underlined TFs, miRNAs and lncRNAs indicate consistency of the target genes up**-** or down-regulated by these common factors between the in vivo atrial or ventricular CMs and hPSC-CMs. Among heart disease, HF = heart failure, arr = arrhythmia and cmp = cardiomyopathy. The boxes in red and green indicate up- and down-regulated target genes, respectively. **C** A proposed model of dynamic development of in vitro hPSC-CMs and in vivo embryo-CMs. Rectangle boxes show the associated biological processes and pathways of the target genes. Those in red indicate processes and pathways whose targets are up-regulated ↑, those in green are down-regulated ↓, and those in gray are both up- and down-regulated genes

Next, we compared the *heart targets* from in vivo CMs to in vitro CMs. We identified target genes consistently up-regulated among CM-AE, CM-VE and the hPSC-CMs at early stages (Fig. 5A), including *CASO2*, *CAV2*, *COL1A1*, *HRC*, *MYL9*, *RYR2*, *TGFBR3* and *TNNI3.* Atrial genes *MYL7*, *TBX5*, *KCNA5* and other cardiac genes like *TNNT2*, *CD36*, *NPPA*, *TTN* and *TRDN* were up-regulated in both CM-AE and hPSC-CMs. Conservation between CM-VE and hPSC-CMs are also evident, with ventricular-prevalent genes such as *MYH7* up-regulated in both. However, some divergence was noted between in vitro and in vivo samples, with ventricular-prevalent K^+^ channel gene *KCNQ1* [37] being up-regulated in CM-VE, but differentially regulated in the various hPSC-CMs. HEY2, known to promote ventricular and repress atrial identities, was reduced in CM-AEs as would be expected, but its expression was divergently regulated in different hPSC-CM datasets. These differences may represent dysfunctional regulatory mechanisms that impede hPSC-CM development or maturation. Significant divergence was found between in vitro and in vivo samples during late stages (Fig. 5B). Most of the *heart targets* are elevated in late relative to early-stage hPSC-CMs but are suppressed or largely unchanged in late-stage atrial and ventricular CMs. Examples include *CKM*, *TNNC1* and *TNNI3.* Some are specifically reduced in CM-VL (*KCNQ1*, *MYL3* and *MYL4*) or CM-VL (*DES*, *MYL7*, *MYL2*, *NPPA*, *TNNT2* and *PLN*).

While it is possible that our direct comparisons of early and late hPSC-CMs vs atrial or ventricular CMs may not be developmentally equivalent, the differences we identified between hPSC and embryo-derived samples may be critical for better understanding why hPSC-CMs fail to adequately mature in vitro relative to in vivo derived CMs. Noticeably, most target genes involved in apoptosis, cell cycle & proliferation and transmembrane transports are not conserved. They are co-regulated during the early stages, likely through interactions of TFs (GATA6, NR2F2, HAND1 and HEY2), miRNAs (miR-133a, -199a, -590 and -499a) or lncRNA (GAS5 and MALAT1) (Table 4). During the late stages, however, the principal regulators include different sets of TFs (SRF, NR2F2, TBX5 and HEY2), miRNAs (miR-1, -133a, -199a and -200c) and lncRNAs (PVT1, MALAT1 and XIST) (Table 4). Moreover, *CD36*, which we have described as a marker for maturation in hPSC-CMs and which is known to be important for heart function [38], is up-regulated in both CM-AL or CM-VL (Fig. 5B). Interestingly, most of the cardiac targets identified through these analyses are associated with heart disease, particularly heart failure, arrhythmia and cardiomyopathy (Fig. 5A, B), underscoring the potential involvement of our chosen regulators in regulating cardiac pathogenesis.

**Table 4:** Specific pathways and biological processes co-regulated by TF-lncRNA-miRNA during development of embryo-derived arial and ventricular cardiomyocytes

Processes/pathway	Embryo-CMs	Main regulatory factors		
		TF	miRNA	lncRNA
Apoptosis	CM-AE	GATA6, HEY2	miR-199a, -590	GAS5, PVT1, MEG3
	CM-VE	HEY2, NR2F2, TBX5, HAND1	miR-499a, -133a	GAS5, MALAT1
	CM-AL	SRF, TBX5, NR2F2	miR-200c	MALAT1, PVT1, XIST, MEG3
	CM-VL	SRF, NR2F2, HEY2	miR-499a, -133a	MALAT1
Cell cycle	CM-AE	HEY2, GATA6	miR-499a, -133a, -590	PVT1, GAS5
	CM-VE	NR2F2, MESP1	miR-199a, -499a, -590	GAS5, H19
	CM-AL	HEY2, HAND1, SRF, NKX2-5, TBX5, NR2F2	miR-200c, -199a	
	CM-VL	MESP1, SRF, NR2F2	miR-199a, -499a, -133a	MIAT, MALAT1
Cell proliferation	CM-AE	GATA6, SRF	miR-590, -23, -199a	MALAT1
	CM-VE	NR2F2, HAND1	miR-590, -133a	GAS5, MALAT1
	CM-AL	TBX5, GATA6	miR-200c, -1	PVT1, MALAT1
Transmembrane transport	CM-VL	GATA6, NR2F2	miR-1	MALAT1

To explore a more comprehensive transcriptional and posttranscriptional regulatory landscape of cardiac differentiation and maturation, we combined all the *heart targets* of the TFs, lncRNAs and miRNAs in human embryo-CMs and hPSC-CMs, and propose a dynamic picture of hierarchical regulatory mechanisms underlying the cross talk among the TFs, ncRNAs and targets and the associated signaling pathways and biological processes (Fig. 5C). These results are based on the highly conserved co-regulatory networks of TF-ncRNA identified to account for the control of transcription during in vitro hPSC differentiation and in vivo cardiac development. We propose that this integrated data analysis using CGRM readily assembles information of ncRNAs with cardiac TFs to infer conserved transcriptional and posttranscriptional regulatory modules that are likely cell autonomous as well as predict how and when differences between in vitro and in vivo development over time occur in human CMs that may explore developmental blocks in vitro.

## Discussion

To unravel highly complex or divergent regulatory mechanisms underlying in vitro versus in vivo heart development and function, we introduce the CGRM framework that performs sophisticated integration and computational modeling of time series biological data. In this study, we focused on in vitro differentiated hPSC-CMs at two temporal stages, and then we compared these data with published datasets obtained from in vivo derived cardiac samples. To accomplish this, CGRM incorporates two gene regulatory models, LogicTRN and RCA. LogicTRN reliably and robustly captures the most likely TF-TF regulation of transcriptional dynamics [32]. RCA modeling captures the sparse structure in expression profiles and infers gene regulatory programs through matrix factorization [33]. As a partial validation of our approach, LogicTRN and RCA have been used to identify gene regulatory programs underlying progression of different cancer in human [32, 33]. CGRM, which utilizes time series expression profile data from mRNAs and various regulator–target data, is capable of decoding potential complex relationships over time among multiple regulatory factors and targets from complex biological systems.

Currently, the web server TIMEOR displays cause-and-effect modeling to integrate time series and multi-omics data for inferring temporal dynamics between TFs [31]. This approach identifies potential TFs that directly interact to form a TF-target network by analyzing DEGs and coupling predicted and observed TF-binding data. Although TIMEOR is used to determine how key regulatory targets interact with each other over time, it does not efficiently integrate different types of data, especially gene expression and regulator–target relationships. An ordinary differential equations-based modeling couples changed levels of mRNA expression with time points during the differentiation of multipotential hematopoietic progenitors [39]. This method infers the type and strength of regulatory interconnections and dynamics of GRNs including key TFs and cytokine receptors; however, it or TIMEOR does not construct multi-layered regulatory networks. CGRM addresses these limitations and considers the complex interactions among TFs, miRNAs and lncRNAs to cooperatively regulate heart development and function. To ensure optimal efficiency of CGRM in prediction of the putative targets generated from the modeling, LogicTRN can identify and prioritize unique TF regulatory logics, which refer to high potential and regulated targets. RCA identifies the most likely targets of regulators using the permutation test to evaluate the significance of results from output matrices. Thus, CGRM allows users to identify or confirm transcriptional controls of gene regulation during cardiac development.

Our results are consistent with a strong and conserved regulatory role of MESP1 in initiating transcriptional regulation of CM differentiation at an early stage, as well as their related pathways or functional processes, supporting conclusions drawn from previous biological experiments [3, 4]. Comparatively, unlike TFs, the roles of ncRNAs are less well characterized. Each ncRNA can potentially regulate a certain number of targets with redundancies, which makes co-regulation even more critical to control transcript levels. Here, we constructed the GRN linking the cardiac TFs and selected miRNAs in early hPSC-CMs. Our results show cooperation among miR-1, -133, -208 and -499 to contribute to early differentiation of CMs [40, 41]. At late stages, our modeling identified miR-200c to be one of the main regulators, and this is supported by our previous study regarding the inhibition of miR-200c on Ca^2+^, K^+^ and Na^+^ transport and handling, contraction and heart function [42]. Consistently miR-1, which is widely reported to activate cardiac differentiation, has been shown experimentally to regulate *KCNJ2* and *GJA1* [43], and cardiac TF *MEF2A*, *MEF2C*, *MEIS1*, *GATA4* and *HAND2* [44–47]. LncRNAs are the most diverse and heterogeneous class of ncRNAs. There have been many reports demonstrating involvement of lncRNAs in CM differentiation and maturation, and disease [9, 11, 19, 20, 48]. Our results indicate the importance of NEAT1 and MALAT1 in hESC/hiPSC-CM differentiation, and of GAS5, H19, MALAT1 and MEG3 in fetal heart development. In particular, NEAT1, H19 and MEG3 regulate many cardiac miRNAs in the early hPSC-CMs. Noticeably, these lncRNAs could work with cardiac TFs to co-regulate heart target genes consistently in both in vitro and in vivo CMs. Although our computational prediction highlights important regulatory roles of lncRNA in both cardiac differentiation and development, the overall picture of lncRNA regulatory function is divergent. Nevertheless, the lncRNAs and the target gene programs identified in this study may provide a guide for experimental examination and verification.

As potential limitations, in vitro studies had different cultivation conditions among three datasets, with one involving lactate selection to eliminate non-CMs. Despite these differences, CGRM identified conserved transcriptional regulatory program underlying cardiac differentiation during early stages of differentiation. Conversely, late-stage regulatory activity is less conserved among the three hPSC-CM datasets, particularly for the lncRNAs and miRNAs. Thus, these culture differences might be reflected in the transcriptome of these cells; however, it is also possible that these divergent factors may contribute to the “developmental” block observed in hPSC-derive CMs. It is therefore noteworthy that most of the functional studies involving hPSC-CMs are performed using CMs at late stages when heterogeneity is known to be a crucial problem in the use of hPSC-CMs [49, 50]. Our study thus also highlights factors that might be inconsistently regulated among different protocols/studies, including ncRNAs, which may shed light on the issue of culture heterogeneity.

We compared our in vitro differentiation in hPSC-CM samples with publicly available data using CMs isolated from human fetal embryos. When compared, the greatest degree of conservation was detected from comparisons of early staged human cells. However, some differences were observed. Unlike in hPSC-CMs, MESP1 does not appear to be a driver TF during the “early stage” CMs obtained during fetal cardiac development, i.e., in CM-AE and CM-VE, suggesting that the “early stages” are not equivalent between the in vitro and in vivo datasets. Our early-stage in vitro data involve days 0–15 of differentiation and cover mesodermal and cardiac commitment, when MESP1 is known to be a critical regulator. Conversely, our earliest time-point in vivo is the 5^th^ week, when CMs are already committed, and MESP1 may be dispensable. Instead, we find that TFs known to control ventricular and atrial specification such as HEY2, NR2F2 and TBX5 can regulate more DEGs during the “early” in vivo derived samples (CM-VE and CM-AE) than in late staged samples (CM-VL and CM-AL), indicating their importance for commitment to the ventricular/atrial fate during the early stages.

A significant finding from this study is the pronounced regulatory dichotomy observed between the regulatory programs from in vitro and in vivo CM samples during later stages. Our data show that the two sample sets share relatively few common heart targets. Of note were genes involved in apoptosis, cell cycle and proliferation and transmembrane transport, which are particularly divergent. Our analysis further indicates that the regulation of cardiac miRNAs and lncRNAs on in vivo specific targets in these processes and pathways is highly divergent [9]. However, some transcripts like CD36, which is important for cardiac fatty acid metabolism, is consistently up-regulated on both in vitro and in vivo CMs. CD36 in hPSC-CMs and independent of differentiation protocol acts as a marker of cardiac maturation [49]. Current analysis again confirms CD36 as a target of regulatory programs that govern both in vitro and in vivo development [38]. This finding is in agreement with results from our laboratory and others that in vitro derived hPSC-CMs do not fully mature nor do they fully recapitulate the in vivo phenotype [38].

## Conclusions

Our findings indicate that CGRM represents an innovative tool for studying the dynamic gene regulation underlying heart development. It is particularly well suited to identify distinct regulatory programs of cardiac TFs, miRNAs and lncRNAs that are predicted to modulate cardiac differentiation and development. By identifying those complex regulatory networks that diverge between in vitro systems and in vivo derived cells, the insights gained from this framework are potentially important for basic and applied studies of hPSC-CMs. By identifying divergent pathways, approaches can be used to drive in vitro maturation, which will be of benefit to pharmacological testing and potential therapeutic applications. Specifically, use of this framework should prove valuable to the development of testable hypotheses designed to identify conserved and divergent pathways that may account for differences among hPSC-CMs that fail to mature as their in vivo counterparts. The web interface also provides users the cardiac data resources so that they have the option of submitting the existing data or providing self-defined data. The workflow of CGRM produces output tables, which can be downloaded for further analyses of regulator interaction, heart function, disease genes and so forth. In conclusion, this is the first web-based framework that can search for cardiac gene regulatory modeling to uncover dynamic transcriptional gene regulation in a high-throughput manner. Although this proof-of-principle study focused on the heart, CGRM can acts as a general framework that allows the integrated analysis of diverse biological systems.

### Supplementary Information

**Additional file 1. **Supplementary information of regulator-target data of TFs, miRNAs and lncRNAs and resources provided at CGRM.

**Additional file 2. Supplementary tables. Table S1: **Target genes of TFs, lncRNAs and miRNAs during the early and late stages of H7-ESC-derived cardiomyocytes, with mRNA expression data extracted from NCBI GSE76523 and our produced data (GSE239918).** Table S2: **Target genes of TFs, lncRNAs and miRNAs during the early and late stages of hiPSC-derived cardiomyocytes, with mRNA expression data extracted from NCBI GSE35671.** Table S3: **Target genes of TFs, lncRNAs and miRNAs during the early and late stages of hiPSC-derived cardiomyocytes, with mRNA expression data extracted from NCBI GSE81585.** Table S4: **The top 50 predicted logics formed by TFs and the regulated genes during the early and late stages of hESC/hiPSC-derived cardiomyocytes.** Table S5:  **Heart target genes co-regulated by cardiac TFs and miRNAs on hESC/hiPSC CM differentiation.** Table S6:  **Heart target genes co-regulated by cardiac TFs and lncRNAs on hESC/hiPSC CM differentiation.** Table S7: **Heart target genes of TFs, lncRNAs and miRNAs in human fetal embryo-developed atrial cardiomyocytes using single cell RNA-seq data (GSE106118).** Table S8: **Heart target genes of TFs, lncRNAs and miRNAs in human fetal embryo-developed ventricular cardiomyocytes using single cell RNA-seq data (GSE106118).** Table S9: **The top 25 predicted logics formed by TFs and the regulated genes during fetal embryo-derived atrial and ventricular CM development.** Table S10: **The predicted heart target genes of TFs, miRNAs and lncRNAs from fetal embryo-derived atrial and ventricular CM development.

**Additional file 3. Supplementary figures. Figure S1: **Enrichment analysis of overlaps between the target genes of the cardiac TFs and of miRNAs during early and late stages of hESC-CM/hiPSC-CM differentiation.** Figure S2: **Enrichment analysis of overlaps between the target genes of the cardiac TFs and of lncRNAs during early and late stages of hESC-CM/hiPSC-CM differentiation.

## Data Availability

The processed or raw count data of mRNA expression shown in Table 1 were downloaded from GEO/NCBI. Our RNA-seq data of H7-CM differentiation with days 15, 30, 45 and 60 are available with GSE239918 (https://www.ncbi.nlm.nih.gov/geo/query/acc.cgi?acc=GSE239918).

## Abbreviations

TF: Transcription factor
miRNA: MicroRNA
lncRNA: Long noncoding RNAs
ncRNA: Noncoding RNA
CGRM: Cardiac gene regulatory modeling
CM: Cardiomyocyte
hPSC: Human pluripotent stem cell
hiPSC: Human-induced pluripotent stem cell
hESC: Human embryonic stem cells
CM-AE: Atrial cardiomyocyte at early stages
CM-AL: Atrial cardiomyocyte at late stages
CM-VE: Ventricular cardiomyocyte at early stage
CM-VL: Ventricular cardiomyocyte at late stages
GRN: Gene regulatory network
TIMEOR: Trajectory Inference and Mechanism Exploration with Omics data in R
RCA: Regulatory component analysis
DEG: Differentially expressed gene
GEO: Gene Expression Omnibus
NCBI: National Center for Biotechnology Information
