# Supplementary material for: Integrated modeling framework reveals co-regulation of transcription factors, miRNAs and lncRNAs on cardiac developmental dynamics
Source: Stem Cell Res Ther. 2023 Sep 13;14:247. doi: 10.1186/s13287-023-03442-0 (PMC10500942; doi:10.1186/s13287-023-03442-0)
Supplement: Supplementary file 1 — Additional file 1. Supplementary information of regulator-target data of TFs, miRNAs and lncRNAs and resources provided at CGRM. [file 13287_2023_3442_MOESM1_ESM.docx]

**Additional file 1**

**Regulator-target data of TFs, miRNAs and lncRNAs**

To run the model systems of the platform, the input data regulator (TFs, miRNAs and lncRNAs)-target (mRNAs or miRNAs) were prepared. According to previous studies, we defined three sets of cardiac regulators which is critical in heart development and function.

***TFs***

The fourteen TFs MESP1, GATA4, GATA6, HAND1, HAND2, ISL1, IRX4, HEY2, MEF2A, MEF2C, NKX2-5, NR2F2, SRF and TBX5 have been widely recognized as critical factors during heart development in both hPSC-CM differentiation and embryo-development [1-4].

***miRNAs***

miRNAs play a central role in cardiogenesis and heart function, including cardiac-specific or cardiac-enriched miR-1, miR-133a, miR-208 and miR-499, that are involved in differentiation and maturation of heart myocytes. Among them, miR-1 regulates differentiation, proliferation [5] and electrophysiological maturation [6] of human derived CMs. miR-133a was found to promote cardiogenic differentiation by targeting epidermal growth factor receptor [7]. While miR-1 and miR-133a are involve in commitment of cardiac-specific muscle lineage from stem cells and mesodermal precursors, miR-208 and miR-499 play an important role in the differentiation of cardioblasts to CMs [8]. In addition, miR-23 [9], miR-199a [10], miR-200c [11], miR-590 [12, 13] and miR-15b [14] also regulate the process of CM differentiation by targeting different genes or transcription factor, respectively. miR-34a regulates cardiac contractile function by targeting PNUTS has been well demonstrated by Boon et al [15]. Other miRNAs, such as let-7i [16], miR-29a [17] and miR-31a [18] were reported related with CM proliferation, even though some miRNAs (e.g. let-7 family) are required for the maturation of stem cell-derived CMs [19]. miRNA also play pivotal roles in pathogenesis of cardiovascular systems, such as miR-1, -133, 208, -25, -223, -31 and -199 in myocardial infarction, hypertrophy, heart failure, arrhythmia, etc. [20, 21]. Studies on miRNA effects and the miRNA-based therapies provide an insight into widespread clinical use of cardiac miRNAs.

***lncRNAs***

LncRNAs is another class of ncRNAs that regulate genetic networks, finally govern CM fate and mediate cardiac regeneration. GAS5 was found involved in CM differentiation of human induced pluripotent stem cells [22], H19 is involved in a wide range of biological processes of cardiogenesis and function, such as CM differentiation [23], proliferation [24] and heart development [25]. MEG3 plays important role in cardiac diastolic dysfunction [26] and cardiac apoptosis [27]. MALAT1 participates in CM electrophysiology [28], proliferation [29], as well as apoptosis [29]. Other lncRNAs, such as MIAT [30], TUG1 [31], XIST [32], PVT1 [33, 34] and NEAT1 [35], have also been reported to be involved in CM apoptosis.

To obtain lncRNA-targeted mRNA or miRNA data, we used three resources: 1) LncBases, containing miRNA targets of lncRNAs, supported by low- and high-throughput, direct or indirect miRNA-lncRNA experimental results [36, 37]; 2) NPInter databases (<http://bigdata.ibp.ac.cn/npinter>) containing interactions of ncRNAs, including lncRNA-mRNAs and lncRNA-miRNAs, supported by experimentally validated and high-throughput experimental data [38]; and 3) LncTarD (<http://biocc.hrbmu.edu.cn/LncTarD>), providing a comprehensive resource of key lncRNA-target regulations and lncRNA-mediated regulatory relations with human diseases [39].

We extracted miRNA-targeted mRNA data from two sources: 1) mirTarBase, a database of miRNA target genes supported by experimental validation [40, 41]; and 2) miRecords, a web-based platform, connecting the predicted binding target genes/mRNAs of miRNAs based on 11 computational methods [42]. Genes or mRNAs were considered binding targets of a particular miRNA if they were identified by at least four methods.

To predict whether a gene is regulated by a TF, we identified the binding sites of TFs using PWMSCAN [43]. This method conducts computational identification of binding sites by scanning promoter sequences using Position Weight Matrices of TF binding motifs. The predicted binding sites were evaluated by calculation of p values via the permutation-based method FastPval [44]. The putative binding sites were filtered based on conservation scores between human and mouse genomes. Promoters were limited to locations 2000 bp upstream and 500 bp downstream from the transcriptional start sites.

**Heart data resource**


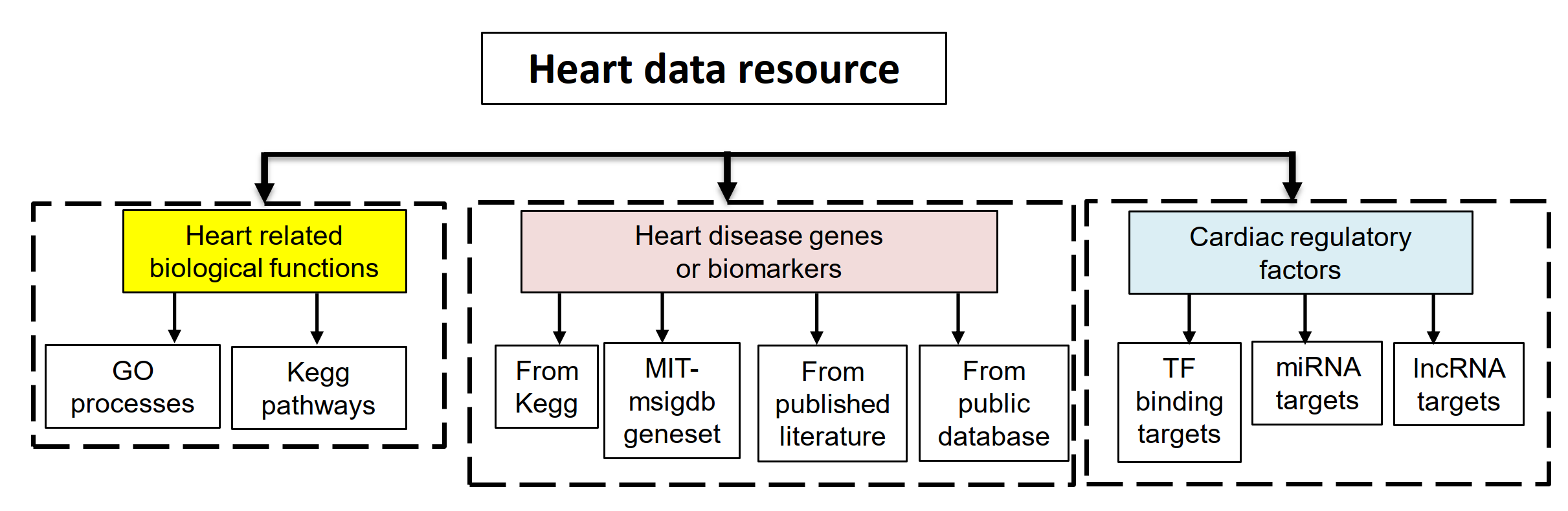
Here, we provide “Resource” web page that that allows researchers for searching and querying a list of input genes/proteins through selection of the query heart related data. This flexibility allows researchers to search for possible molecular interactions or association among input genes/proteins/miRNAs and these provided resources (**Figure above**). Three types of data can be available, 1) heart-related biological processes and pathways; 2) heart disease genes or biomarkers; 3) cardiac regulatory targets of transcription factors (TFs), miRNAs and lncRNAs derived from experimental, publications and computational analysis. Using software and statistical tool in “Interaction and Association”, we can identify whether the input genes/mRNAs/miRNAs/lncRNAs can be detected with these data resources, and how significant enrichment of between these input molecules among these gene sets provided by the heart data resources. The output display is in tabular form and downable and provides merged table showing.

**References**

1. Kathiriya IS, Nora EP, Bruneau BG: **Investigating the transcriptional control of cardiovascular development.** *Circ Res* 2015, **116:**700-714.

2. Bondue A, Blanpain C: **Mesp1: a key regulator of cardiovascular lineage commitment.** *Circ Res* 2010, **107:**1414-1427.

3. Rojas-Munoz A, Maurya MR, Lo F, Willems E: **Integrating omics into the cardiac differentiation of human pluripotent stem cells.** *Wiley Interdiscip Rev Syst Biol Med* 2014, **6:**311-328.

4. Cerutti C, Bricca G, Rome S, Paultre CZ, Gustin MP: **Robust coordination of cardiac functions from gene co-expression reveals a versatile combinatorial transcriptional control.** *Mol Biosyst* 2014, **10:**2415-2425.

5. Sluijter JP, van Mil A, van Vliet P, Metz CH, Liu J, Doevendans PA, Goumans MJ: **MicroRNA-1 and -499 regulate differentiation and proliferation in human-derived cardiomyocyte progenitor cells.** *Arterioscler Thromb Vasc Biol* 2010, **30:**859-868.

6. Fu JD, Rushing SN, Lieu DK, Chan CW, Kong CW, Geng L, Wilson KD, Chiamvimonvat N, Boheler KR, Wu JC, et al: **Distinct roles of microRNA-1 and -499 in ventricular specification and functional maturation of human embryonic stem cell-derived cardiomyocytes.** *PLoS One* 2011, **6:**e27417.

7. Lee SY, Ham O, Cha MJ, Song BW, Choi E, Kim IK, Chang W, Lim S, Lee CY, Park JH, et al: **The promotion of cardiogenic differentiation of hMSCs by targeting epidermal growth factor receptor using microRNA-133a.** *Biomaterials* 2013, **34:**92-99.

8. Chistiakov DA, Orekhov AN, Bobryshev YV: **Cardiac-specific miRNA in cardiogenesis, heart function, and cardiac pathology (with focus on myocardial infarction).** *J Mol Cell Cardiol* 2016, **94:**107-121.

9. Lu M, Xu Y, Wang M, Guo T, Luo F, Su N, Wang Z, Xu L, Liu Z: **MicroRNA-23 inhibition protects the ischemia/reperfusion injury via inducing the differentiation of bone marrow mesenchymal stem cells into cardiomyocytes.** *Int J Clin Exp Pathol* 2019, **12:**1060-1069.

10. Chen HP, Wen J, Tan SR, Kang LM, Zhu GC: **MiR-199a-3p inhibition facilitates cardiomyocyte differentiation of embryonic stem cell through promotion of MEF2C.** *J Cell Physiol* 2019, **234:**23315-23325.

11. Poon EN, Hao B, Guan D, Jun Li M, Lu J, Yang Y, Wu B, Wu SC, Webb SE, Liang Y, et al: **Integrated transcriptomic and regulatory network analyses identify microRNA-200c as a novel repressor of human pluripotent stem cell-derived cardiomyocyte differentiation and maturation.** *Cardiovasc Res* 2018, **114:**894-906.

12. Wang F, Zhang H, Wang C: **MiR-590-3p regulates cardiomyocyte P19CL6 proliferation, apoptosis and differentiation in vitro by targeting PTPN1 via JNK/STAT/NF-kB pathway.** *Int J Exp Pathol* 2020, **101:**196-202.

13. Ekhteraei-Tousi S, Mohammad-Soltani B, Sadeghizadeh M, Mowla SJ, Parsi S, Soleimani M: **Inhibitory effect of hsa-miR-590-5p on cardiosphere-derived stem cells differentiation through downregulation of TGFB signaling.** *J Cell Biochem* 2015, **116:**179-191.

14. Zhao MJ, Xie J, Shu WJ, Wang HY, Bi J, Jiang W, Du HN: **MiR-15b and miR-322 inhibit SETD3 expression to repress muscle cell differentiation.** *Cell Death Dis* 2019, **10:**183.

15. Boon RA, Iekushi K, Lechner S, Seeger T, Fischer A, Heydt S, Kaluza D, Tréguer K, Carmona G, Bonauer A, et al: **MicroRNA-34a regulates cardiac ageing and function.** *Nature* 2013, **495:**107-110.

16. Hu Y, Jin G, Li B, Chen Y, Zhong L, Chen G, Chen X, Zhong J, Liao W, Liao Y, et al: **Suppression of miRNA let-7i-5p promotes cardiomyocyte proliferation and repairs heart function post injury by targetting CCND2 and E2F2.** *Clin Sci (Lond)* 2019, **133:**425-441.

17. Cao X, Wang J, Wang Z, Du J, Yuan X, Huang W, Meng J, Gu H, Nie Y, Ji B, et al: **MicroRNA profiling during rat ventricular maturation: A role for miR-29a in regulating cardiomyocyte cell cycle re-entry.** *FEBS Lett* 2013, **587:**1548-1555.

18. Xiao J, Liu H, Cretoiu D, Toader DO, Suciu N, Shi J, Shen S, Bei Y, Sluijter JP, Das S, et al: **miR-31a-5p promotes postnatal cardiomyocyte proliferation by targeting RhoBTB1.** *Exp Mol Med* 2017, **49:**e386.

19. Kuppusamy KT, Jones DC, Sperber H, Madan A, Fischer KA, Rodriguez ML, Pabon L, Zhu WZ, Tulloch NL, Yang X, et al: **Let-7 family of microRNA is required for maturation and adult-like metabolism in stem cell-derived cardiomyocytes.** *Proc Natl Acad Sci U S A* 2015, **112:**E2785-2794.

20. Wojciechowska A, Braniewska A, Kozar-Kaminska K: **MicroRNA in cardiovascular biology and disease.** *Adv Clin Exp Med* 2017, **26:**865-874.

21. Barwari T, Joshi A, Mayr M: **MicroRNAs in Cardiovascular Disease.** *J Am Coll Cardiol* 2016, **68:**2577-2584.

22. Ye D, Bao Z, Yu Y, Han Z, Yu Y, Xu Z, Ma W, Yuan Y, Zhang L, Xu Y, et al: **Inhibition of cardiomyocyte differentiation of human induced pluripotent stem cells by Ribavirin: Implication for its cardiac developmental toxicity.** *Toxicology* 2020, **435:**152422.

23. Ragina NP, Schlosser K, Knott JG, Senagore PK, Swiatek PJ, Chang EA, Fakhouri WD, Schutte BC, Kiupel M, Cibelli JB: **Downregulation of H19 improves the differentiation potential of mouse parthenogenetic embryonic stem cells.** *Stem Cells Dev* 2012, **21:**1134-1144.

24. Afify ARY: **The long non-coding road to endogenous cardiac regeneration.** *Heart Fail Rev* 2019, **24:**587-600.

25. Viereck J, Bührke A, Foinquinos A, Chatterjee S, Kleeberger JA, Xiao K, Janssen-Peters H, Batkai S, Ramanujam D, Kraft T, et al: **Targeting muscle-enriched long non-coding RNA H19 reverses pathological cardiac hypertrophy.** *Eur Heart J* 2020, **41:**3462-3474.

26. Piccoli MT, Gupta SK, Viereck J, Foinquinos A, Samolovac S, Kramer FL, Garg A, Remke J, Zimmer K, Batkai S, Thum T: **Inhibition of the Cardiac Fibroblast-Enriched lncRNA Meg3 Prevents Cardiac Fibrosis and Diastolic Dysfunction.** *Circ Res* 2017, **121:**575-583.

27. Wu H, Zhao ZA, Liu J, Hao K, Yu Y, Han X, Li J, Wang Y, Lei W, Dong N, et al: **Long noncoding RNA Meg3 regulates cardiomyocyte apoptosis in myocardial infarction.** *Gene Ther* 2018, **25:**511-523.

28. Zhu P, Yang M, Ren H, Shen G, Chen J, Zhang J, Liu J, Sun C: **Long noncoding RNA MALAT1 downregulates cardiac transient outward potassium current by regulating miR-200c/HMGB1 pathway.** *J Cell Biochem* 2018, **119:**10239-10249.

29. Li L, Wang Q, Yuan Z, Chen A, Liu Z, Wang Z, Li H: **LncRNA-MALAT1 promotes CPC proliferation and migration in hypoxia by up-regulation of JMJD6 via sponging miR-125.** *Biochem Biophys Res Commun* 2018, **499:**711-718.

30. Chen L, Zhang D, Yu L, Dong H: **Targeting MIAT reduces apoptosis of cardiomyocytes after ischemia/reperfusion injury.** *Bioengineered* 2019, **10:**121-132.

31. Jiang N, Xia J, Jiang B, Xu Y, Li Y: **TUG1 alleviates hypoxia injury by targeting miR-124 in H9c2 cells.** *Biomed Pharmacother* 2018, **103:**1669-1677.

32. Cai CL, Jin L, Lang XL, Li BL: **Long noncoding RNA XIST regulates cardiomyocyte apoptosis by targeting miR-873-5p/MCL1 axis.** *Eur Rev Med Pharmacol Sci* 2020, **24:**12878-12886.

33. Ouyang M, Lu J, Ding Q, Qin T, Peng C, Guo Q: **Knockdown of long non-coding RNA PVT1 protects human AC16 cardiomyocytes from hypoxia/reoxygenation-induced apoptosis and autophagy by regulating miR-186/Beclin-1 axis.** *Gene* 2020, **754:**144775.

34. Xu JJ, Zheng WH, Wang J, Chen YY: **Long non-coding RNA plasmacytoma variant translocation 1 linked to hypoxia-induced cardiomyocyte injury of H9c2 cells by targeting miR-135a-5p/forkhead box O1 axis.** *Chin Med J (Engl)* 2020, **133:**2953-2962.

35. Yan H, Liang H, Liu L, Chen D, Zhang Q: **Long noncoding RNA NEAT1 sponges miR‑125a‑5p to suppress cardiomyocyte apoptosis via BCL2L12.** *Mol Med Rep* 2019, **19:**4468-4474.

36. Karagkouni D, Paraskevopoulou MD, Tastsoglou S, Skoufos G, Karavangeli A, Pierros V, Zacharopoulou E, Hatzigeorgiou AG: **DIANA-LncBase v3: indexing experimentally supported miRNA targets on non-coding transcripts.** *Nucleic Acids Res* 2020, **48:**D101-D110.

37. Paraskevopoulou MD, Georgakilas G, Kostoulas N, Reczko M, Maragkakis M, Dalamagas TM, Hatzigeorgiou AG: **DIANA-LncBase: experimentally verified and computationally predicted microRNA targets on long non-coding RNAs.** *Nucleic Acids Res* 2013, **41:**D239-245.

38. Teng X, Chen X, Xue H, Tang Y, Zhang P, Kang Q, Hao Y, Chen R, Zhao Y, He S: **NPInter v4.0: an integrated database of ncRNA interactions.** *Nucleic Acids Res* 2020, **48:**D160-D165.

39. Zhao H, Shi J, Zhang Y, Xie A, Yu L, Zhang C, Lei J, Xu H, Leng Z, Li T, et al: **LncTarD: a manually-curated database of experimentally-supported functional lncRNA-target regulations in human diseases.** *Nucleic Acids Res* 2020, **48:**D118-D126.

40. Chou CH, Chang NW, Shrestha S, Hsu SD, Lin YL, Lee WH, Yang CD, Hong HC, Wei TY, Tu SJ, et al: **miRTarBase 2016: updates to the experimentally validated miRNA-target interactions database.** *Nucleic Acids Res* 2016, **44:**D239-247.

41. Hsu SD, Tseng YT, Shrestha S, Lin YL, Khaleel A, Chou CH, Chu CF, Huang HY, Lin CM, Ho SY, et al: **miRTarBase update 2014: an information resource for experimentally validated miRNA-target interactions.** *Nucleic Acids Res* 2014, **42:**D78-85.

42. Xiao F, Zuo Z, Cai G, Kang S, Gao X, Li T: **miRecords: an integrated resource for microRNA-target interactions.** *Nucleic Acids Res* 2009, **37:**D105-110.

43. Wang J, Ungar LH, Tseng H, Hannenhalli S: **MetaProm: a neural network based meta-predictor for alternative human promoter prediction.** *BMC Genomics* 2007, **8:**374.

44. Li MJ, Sham PC, Wang J: **FastPval: a fast and memory efficient program to calculate very low P-values from empirical distribution.** *Bioinformatics* 2010, **26:**2897-2899.
