# Supplementary material for: Integrated modeling framework reveals co-regulation of transcription factors, miRNAs and lncRNAs on cardiac developmental dynamics
Source: Stem Cell Res Ther. 2023 Sep 13;14:247. doi: 10.1186/s13287-023-03442-0 (PMC10500942; doi:10.1186/s13287-023-03442-0)
Supplement: Supplementary file 3 — Additional file 3. Supplementary figures. Figure S1: Enrichment analysis of overlaps between the target genes of the cardiac TFs and of miRNAs during early and late stages of hESC-CM/hiPSC-CM differentiation. Figure S2: Enrichment analysis of overlaps between the target genes of the cardiac TFs and of lncRNAs during early and late stages of hESC-CM/hiPSC-CM differentiation. [file 13287_2023_3442_MOESM3_ESM.pdf]

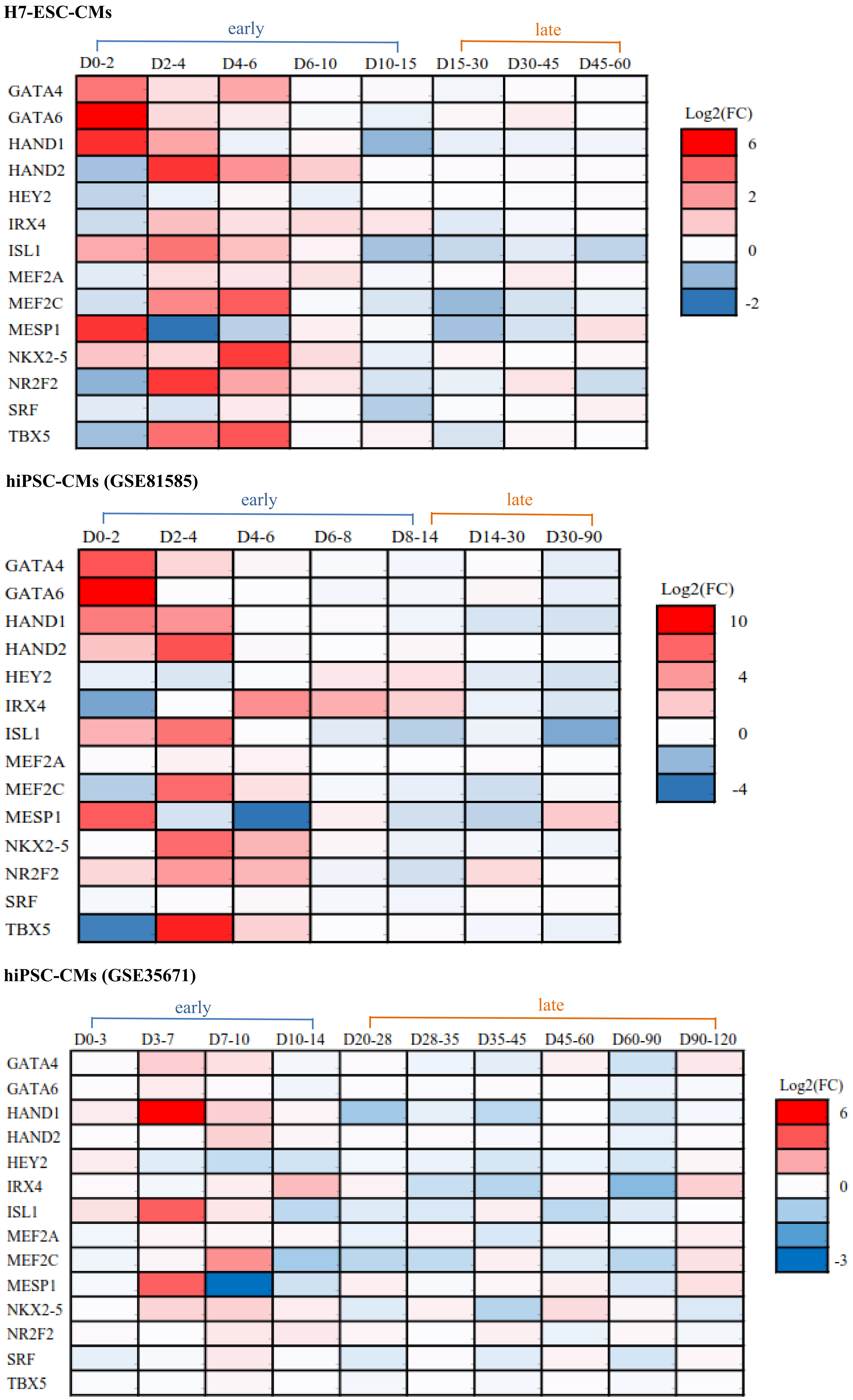

Figure S1. Heatmep of differential expression changes of the cardiac TFs during early and late stages of hESC-CM and hiPSC-CM differentiation.

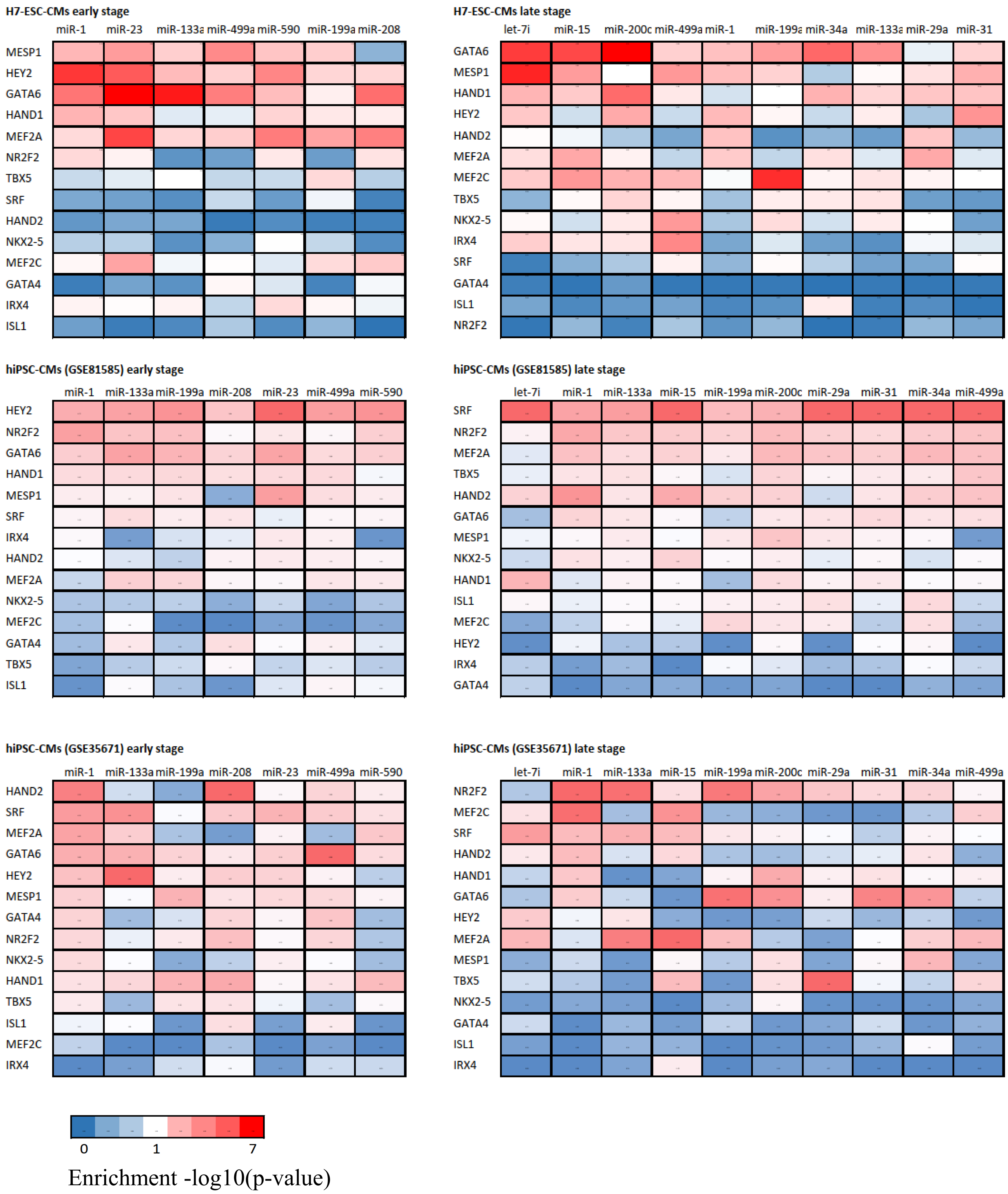

Figure S2. Enrichment analysis of overlaps between the target genes of the cardiac TFs and of miRNAs during early and late stages of hESC-CM/hiPSC-CM differentiation. The p-values account for the significance of the enrichment analysis based on hypergeometric distribution.

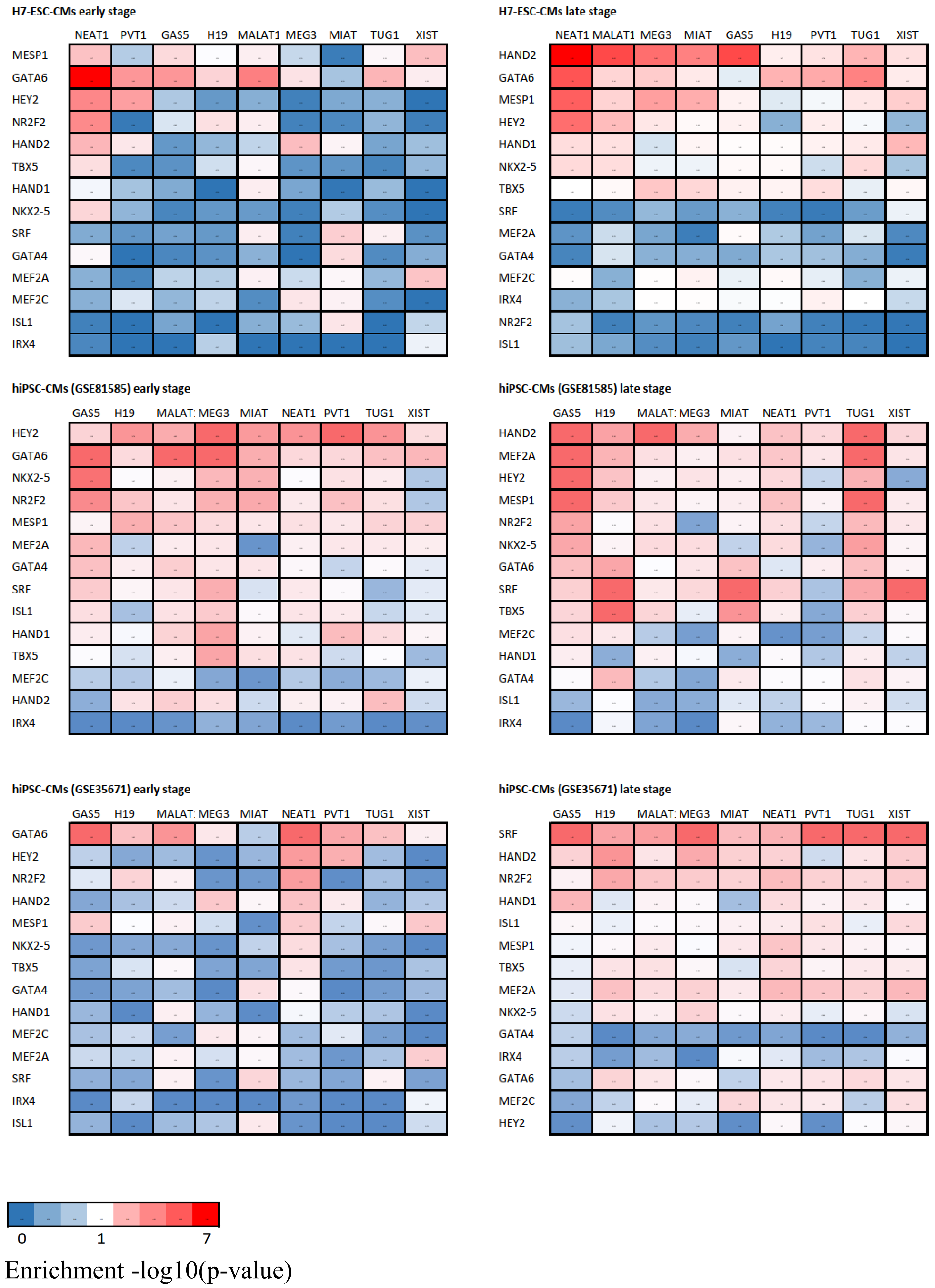

Figure S3. Enrichment analysis of overlaps between the target genes of the cardiac TFs and of lncRNAs during early and late stages of hESC-CM/hiPSC-CM differentiation. The p-values account for the significance of the enrichment analysis based on hypergeometric distribution.
